# Exosome Proteome of U-87MG Glioblastoma Cells

**DOI:** 10.3390/biology5040050

**Published:** 2016-12-06

**Authors:** Sohyun Chun, Seunghyun Ahn, Chang-Hwan Yeom, Seyeon Park

**Affiliations:** 1 Department of Applied Chemistry, Dongduk Women’s University, 60 Hwarang-ro 13-gil, Seongbuk-gu, Seoul 136-714, Korea; www4510@dongduk.ac.kr (S.C.); mistahn321@naver.com (S.A.); 2 Division of Bioscience and Biotechnology, Bio/Molecular Informatics Center, Konkuk University, Seoul 143-701, Korea; 3 Yeom’s Clinic of Palliative Medicine, Seoul 169-410, Korea; lymphych@hanmail.net

**Keywords:** exosome, U-87MG, glioblastoma, proteome, external stimulus

## Abstract

Exosomes are small membrane vesicles between 30 and 100 nm in diameter secreted by many cell types, and are associated with a wide range of physiological and/or pathological processes. Exosomes containing proteins, lipids, mRNA, and microRNA contribute to cell-to-cell communication and cell-to-environment regulation, however, their biological functions are not yet fully understood. In this report, exosomes in the glioblastoma cell line, U-87MG, were isolated and the proteome was investigated. In addition, exosome proteome changes in U-87MG cells exposed to a low temperature were investigated to elucidate whether the exosome proteome could respond to an external stimulus. Cell culture medium was collected, and exosomes were isolated by continuous centrifugation eliminating cell debris, nucleic acids, and other particles. The morphology of exosomes was observed by cryo-tunneling electron microscopy. According to 2-dimensional electrophoresis and matrix-assisted laser desorption ionization time-of-flight mass spectrometry, certain proteins including collagen type VI alpha 1, putative RNA-binding protein 15B chain A, substrate induced remodeling of the active site regulates HTRA1, coatomer protein complex-subunit beta 2, myosin-heavy chain 1, and keratin-type I cytoskeletal 9 showed differences between the control proteome and the low temperature-exposed proteome.

## 1. Introduction

Small vesicles that are released by many cell types can be considered as tools for intercellular communication [1]. These mechanisms may occur via growth factors, cytokines, or small molecular mediators such as hormones, bioactive lipids, nucleotides, and several ions, which exchange information between cells [2,3,4,5]. Most cells shed their membrane-derived microvesicles (MVs) containing various proteins and lipids similar to their cells of origin [1]. In particular, among MVs, exosomes are an active subject of research due to their size and bioactivity, although their biological function has not yet been clarified [6].

Exosomes were first discovered in maturing mammalian reticulocytes, and are small membrane vesicles (30–100 nm) of endocytic origin that are released by a multitude of cell types as a consequence of the fusion of multivesicular bodies (MVBs) (late endosomes, lysosomes) with the plasma membrane [7,8]. Exosomes are secreted from the plasma membrane in two steps: “budding” and “shedding” [9]. They are formed in endocytic compartments (called MVBs) during endosome maturation by inward budding of their limiting membrane, and subsequently secreted into the extracellular milieu [10].

As a consequence of proteins and lipids being sorted at the limiting membrane of endosomes during the formation of intraluminal vesicles (ILVs) in MVBs, exosomes harbor a concrete set of molecules [11,12]. Previous research has shown that exosomes can deliver not only proteins but also mRNA, microRNA, and organelles (e.g., mitochondria), which mediate cell-to-cell communication [1]. They are thought to play a variety of roles such as cell stimulation, delivery of infectious agents into cells (e.g., prions and human immunodeficiency virus) [13,14], and as a biomarker for neurological disease (e.g., Alzheimer’s disease), depending on their origin [15].

According to recent research, exosomes can be harvested from the culture supernatant of several cell types of hematopoietic origin (B cells [16,17], mast cells [18,19], dendritic cells [20], T cells [21], and platelets [22]), as well as cells of non-hematopoietic origin (intestinal epithelial cells [23], Schwann cells [24], tumor cells [25], and neuronal cells [26]). In addition, there is increasing evidence for the presence of exosomes in physiological fluid such as saliva, plasma, breast milk [27,28], malignant and pleural effusions [28], amniotic fluid [29], cerebral spinal fluid [30], sperm [31], and urine [32].

In the present study, the human glioblastoma U-87MG cell line was used to demonstrate the presence of tumor-derived cell exosomes. The U-87MG glioma cell line represents grade IV glioblastoma, which is the most severe form of glioma [33]. The presence of U-87MG cell exosomes were confirmed, along with biochemical and transmission electron microscopy (TEM) evidence. These exosomes share biochemical and biophysical properties with exosomes from other cell types, including size, density, and canonical heat shock protein content [34]. Using Western blotting and gel-based proteomics, we have established both the commonality and uniqueness of the glioblastoma exosome proteome. Herein, cells were exposed to a low temperature of 18 °C for 6 h.

Cell culture medium was collected, and exosomes were isolated from cell debris, nucleic acids, and other large unimportant particles, by continuous centrifugation. The isolated exosomal proteins were analyzed to investigate the differences in expression of exosomal proteins between general conditions and low temperature stress. The morphology of the exosomes was observed using cryo-TEM. The existence of common exosome proteins and their effects on other cells were detected by Western blotting. Exosomal proteins were analyzed by two-dimensional electrophoresis (2-DE) and matrix-assisted laser desorption ionization time-of-flight mass spectrometry (MALDI-TOF-MS). The protein information was found from the NCBInr protein database using the Mascot search engine.

## 2. Materials and Methods

### 2.1. Cell Culture and Treatment

The human glioblastoma cell line, U-87MG, was purchased from the Korean Cell Line Bank (KCLB, Seoul, Korea) and cultured in complete medium: Minimum Essential Medium (MEM; Welgene, Daegu, Korea) supplemented with 10% (*v*/*v*) fetal bovine serum (FBS; Welgene, Daegu, Korea) and 1% antibiotics (10,000 units/mL penicillin, 10 mg/mL streptomycin, and 25 μg/mL amphotericin B) (Welgene, Daegu, Korea). At ~70% confluency (total of four days), the U-87MG cells were separated into two groups for treatment. One group of cells was incubated in a 5% CO_2_ humidified incubator (Thermo Fisher Scientific, Waltham, MA, USA) at 37 °C, and maintained under these conditions. The other group was also incubated under the same conditions, however, was exposed to 18 °C in a low-temperature incubator (Vision Scientific, Daejeon, Korea). During an additional two-day culture, the cells were treated at 18 °C for 30 min, every 12 h. The media was collected.

### 2.2. Isolation and Purification of Exosomes

Each collected fraction of media was centrifuged at 500× *g* at room temperature (Hanil Science Industrial, Gangneung, Korea) for 10 min to eliminate floating particles and dead cells. Pellets were discarded, and the supernatant was again centrifuged at 800× *g* at room temperature for 10 min. At this point, dead cells were collected as a pellet. The collected fractions of supernatant were concentrated to a small volume using the Quixstand benchtop system (GE Healthcare, Buckinghamshire, UK) with a 50 kDa membrane filter (Hollow fiber cartridges; GE Healthcare, Buckinghamshire, UK). Concentrated solution was then centrifuged at 2000× *g* at room temperature for 20 min to remove cell debris and large particles. The resultant supernatants were subsequently centrifuged at 20,000× *g* at 4 °C using a Type 70 Ti rotor (Optima™ L-100 ultracentrifuge; Beckman Coulter, Brea, CA, USA) for 20 min. The pellets contained unimportant particles and vesicles larger than exosomes. The supernatant was centrifuged at 100,000× *g* at 4 °C for 70 min to settle the exosomes [20]. The supernatant was then discarded, and the resulting pellets were washed carefully in 1× phosphate-buffered saline (PBS; Welgene, Daegu, Korea). Resuspended pellets were centrifuged again at 100,000× *g* at 4 °C using a 90 Ti rotor (Beckman Coulter, Brea, CA, USA) for 70 min. Following removal of the supernatant, the pelleted exosomes were resuspended in 100 μL PBS and stored at −80 °C.

### 2.3. Cryo-Transmission Electron Microscopy (TEM)

A volume of 3–5 μL of each exosome sample suspended in PBS was transferred onto a holey carbon-coated grid. The grid was placed at ~85% humidity for 10 s and then plunge-frozen in liquid ethane prepared without ice crystals at liquid-nitrogen temperature (−196 °C) using a Vitrobot (FEI, Hillsboro, OR, USA). The grid was mounted onto a cryo-transfer specimen holder (Gatan, Pleasanton, CA, USA), at −175 °C. The sample was observed using a cryo-TEM (FEI, Hillsboro, OR, USA), operating at 200 kV. Low-dose images were recorded at a nominal magnification of 50,000×.

### 2.4. Two-Dimensional Electrophoresis (2-DE) and Gel Image Analysis

2-DE was carried out as described. Aliquots in sample buffer (7 M Urea, 2 M thiourea, 4.5% (*w*/*v*) 3-[(3-cholamidopropyl)dimethylammonio]-1-propanesulfonate (CHAPS), 100 mM DTT, 40 mM Tris, pH 8.8, and trace of bromophenol blue) were applied to an immobilized pH 3–10 nonlinear gradient strip (Amersham Biosciences, Uppsala, Sweden). The samples (1 mg) were loaded. IEF was performed at 80,000 Vh. Then, 9%–16% linear gradient polyacrylamide gels electrophoresis (18 × 20 cm, 1.5 mm) was performed at a constant 40 mA, for approximately 5 h. The gels were fixed in 40% methanol and 5% phosphoric acid for 1 h and stained with Coomassie Brilliant Blue (CBB) G-250 for 12 h. The gels were destained with water, scanned in a Bio-Rad (Richmond, CA, USA) GS710 densitometer, and then analyzed using the Image Mater Platinum 5.0 image analysis program (Amersham Biosciences).

### 2.5. In-Gel Enzymatic Digestion and MALDI-TOF-MS

For peptide mass fingerprinting (PMF), the spots were excised from the stained gel and in-gel digestion was carried out. The excised spots were destained with 50 mM ammonium bicarbonate in 40% acetonitrile, and dried with a speed vac (Heto Lab Equipment, Allerod, Denmark). The destained spots were rehydrated in 12.5 ng/μL trypsin in 50 mM ammonium bicarbonate. The rehydrated spots were placed on ice for 45 min and treated with 50 mM ammonium bicarbonate (10 μL). The spots were then incubated at 37 °C for 12 h.

For MALDI-TOF-MS analysis, the peptides were desalted and concentrated using a POROS R2 and Oligo R3 column (Applied Biosystems, Fostercity, CA, USA). The column was washed sequentially with 70% acetonitrile, 100% acetonitrile, and 50 mM ammonium bicarbonate. The samples were applied and eluted with cyano-4-hydroxycinnamic acid (CHCA) (Sigma, St. Louis, MO, USA) dissolved in 70% acetonitrile and 2% formic acid onto the MALDI plate (Opti-TOF™ 384-well Insert, Applied Biosystems) [35]. MALDI-TOF-MS was performed on a 4800 MALDI-TOF/TOF™ Analyzer (Applied Biosystems) equipped with a 355-nm Nd:YAG laser (TOF analyzer pressure: approximately 7.6 × 10^−7^ Torr). The mass spectra were obtained in the reflection mode (accelerating voltage of 20 kV and the sum from either 500 laser pulses), and calibrated using the 4700 calibration mixture (Applied Biosystems).

### 2.6. Mascot Database Search

The database search criteria were as follows: taxonomy, *Homo sapiens*; fixed modification, cysteine carboxyamidomethylation; variable modification, methionine oxidation; 1 maximum missed cleavage allowed; 100 ppm MS tolerance.

The probability of the identification of a protein was based on the Mascot score. The protein score is –Log_10_ (P) (P is the probability that the match is observed at a random event). Protein scores greater than 66 are considered significant (*p* < 0.05).

### 2.7. Western Blotting Analysis

To confirm the isolation of exosome, proteins were separated by SDS-PAGE. After the exosome proteins were transferred onto a nitrocellulose membrane (Schleicher and Schuell, Keene, NH, USA), the membrane was blocked with 5% (*w*/*v*) non-fat dry milk in Tris-buffered saline (TBS)–0.1% Tween-20 (TBST) at 4 °C. Then, the membrane was incubated with an anti-HSP90 and an anti-CD63 antibody (Cell Signaling) for 5 h. The membrane was incubated with an anti-rabbit secondary antibody coupled to peroxidase (Cell signaling). After a 2 h-incubation at room temperature, the membrane was washed with TBST, and blots were detected with an enhanced chemiluminescence solution (ECL1; Amersham Biosciences).

## 3. Results

### 3.1. Isolation of Exosomes

The white-yellow pelleted exosomes were isolated from the culture media of both the control and the low temperature-treated U-87MG cells by continuous centrifugation. When the cells were grown to 70% confluency, approximately 16.7 μg exosomes were collected per 75-cm^3^ cell culture flask. It is especially noteworthy that the quantity of exosomes in the medium from cells exposed to low temperature stress was less than that from the non-treated cells.

### 3.2. Identification of Exosomes from U-87MG Cell Culture Medium by Cryo-Transmission Electron Microscopy (TEM)

**Figure 1:**
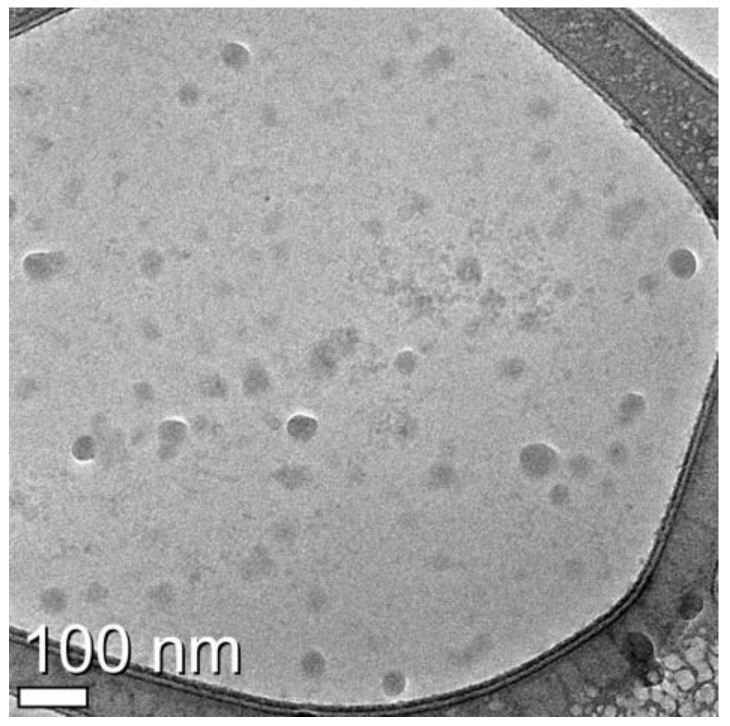
Cryo-transmission electron microscopy (TEM) image of exosomes from U-87MG cell culture medium. Exosomes were isolated by continuous centrifugation. Image was captured of the exosome fraction (suspended in less than 500 μL 1× PBS).

The morphologically-intact vesicles were purified using buoyant density centrifugation. As can be seen, transmission electron microscopy revealed that most of the nanovesicles were small and had a characteristic doughnut morphology (Figure 1). The densitometric analysis showed that the mean diameter of the tumor-derived nanovesicles was 46 nm, which is consistent with the results of a previous study regarding tumor-derived exosomes [25,36].

### 3.3. Protein Analysis of U-87MG Exosomes by Immunoblotting

Recent research has analyzed the protein composition of exosomes refined from various sources, particularly from cell culture medium, by proteomics, Western blotting, and immunocytochemistry. This composition reflects the cell type from which they are released, in addition to the endosomal origin [11].

**Figure 2:**
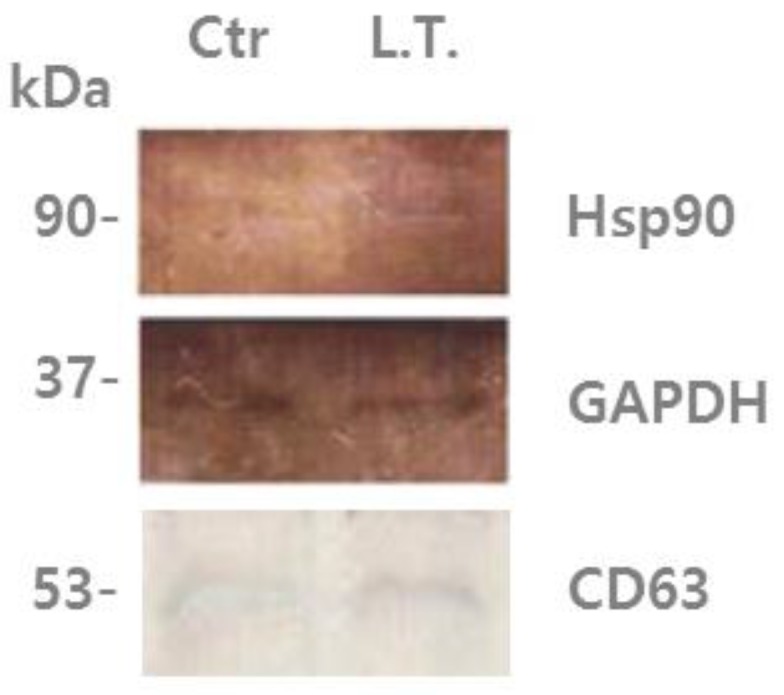
U-87MG exosome proteins were analyzed by immunoblotting using an antibody against Hsp90 and CD63. Left lane: “control”, non-treated U-87MG cell exosomes, which were incubated with 5% CO_2_ at 37 °C for 4 days. Right lane: treated cell (L.T.) exosomes, which were exposed to 18 °C three times for 30 min during incubation with 5% CO_2_ for the last 36 h of the 4-day period.

In the present study, it was found that U-87MG exosomes contain Hsp90 (heat shock protein 90), which is one of the most common heat-related proteins that exist in all branches of eukaryotes. This protein is a highly conserved molecular chaperone that assists other key proteins involved in apoptosis, survival, and growth pathways, to properly fold and stabilize proteins against heat and other environmental stresses [37,38]. The presence of chaperones such as Hsp90 could be driven by their co-sorting with other proteins [11]. In Figure 2, it can be seen that Hsp90 was increased in exosomes from the cells exposed to low temperature (L.T.) compared with those from the non-treated cells. This suggests that when the cells experience an external stimulus, such as a decrease in temperature, the protein composition changes, as well as the protein composition of their exosomes.

### 3.4. Characterization of Exosomal Proteins by Two-Dimensional Polyacrylamide Gel Electrophoresis

**Figure 3:**
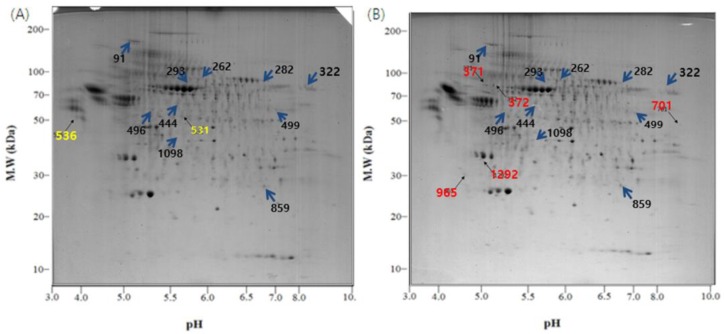
2-DE gel images of the exosome proteome from U-87MG cells. (**A**) Exosomal proteins from U-87MG cells for 2-DE, which were incubated at 37 °C, 5% CO_2_ for 4 days without any change in temperature; (**B**) During the incubation at 37 °C, 5% CO_2_ for 4 days, the cells were exposed to 18 °C three times for 30 min, over a period of 36 h in a low temperature incubator (L.T.). Location of significant protein spots on 2-DE gels was represented as arrows. (blue: Spots that exist on both the control and the L.T. gel but express in different intensity; yellow: Spots that exist only on the control gel; red: Spots that exist only on the L.T. gel).

**Figure 4:**
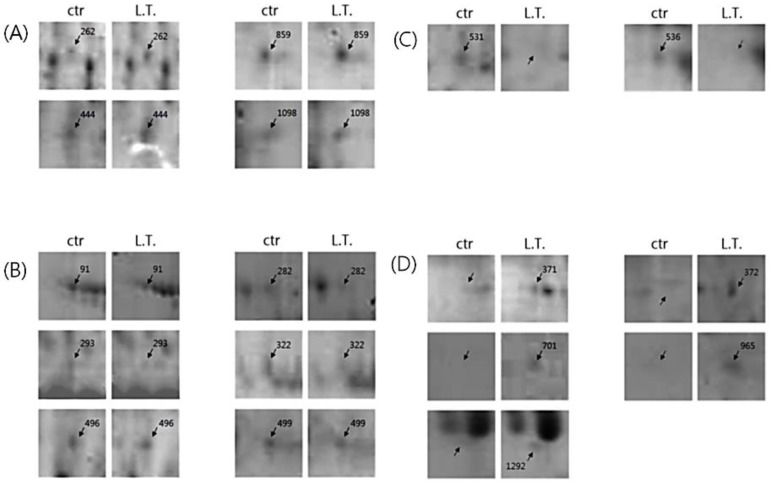
Selected spot images. (**A**) The proteins whose expression was increased in the L.T. gel; (**B**) The proteins whose expression was decreased in the L.T. gel; (**C**) The protein spots that were only present in the control gel; (**D**) The protein spots that were only identified in the L.T. gel.

Of the many spots on the gels, the significant ones were analyzed using the Image Mater Platinum 5.0 image analysis program. There were several different spots between the two temperature conditions (Figure 3 and Figure 4). The spots on the control gel (exosomes from U-87MG cells incubated under normal conditions) were selected, numbered, and compared with spots in the same location on the L.T. gel (exosomes from U-87MG cells exposed to low temperature). Quantitative analysis was then performed. The spots that showed two-fold greater or less expression (% vol of gels) than the control are shown in Table 1. The quantitative differences were only determined when a matched spot showed the same degree of up- or downregulation in duplicate experiments.

### 3.5. Proteome Analysis by MALDI-TOF-MS and Mascot Search

The mass spectra were calibrated using the 4700 calibration mixture (Applied Biosystems). For analysis, the spectrum was internally calibrated with the trypsin peak (842.5 *m*/*z*, 2211.104 *m*/*z*). The meaningful proteins were found from the NCBInr database using the Mascot search engine (Matrix Science) (Table 1). With the input being taxonomy *Homo sapiens*, the protein information corresponding to significant spots was obtained.

There were some meaningful proteins with a protein score higher than 66 (Table 1). In spot No. 444, the protein expression was increased, whereas in spots No. 91, 322, and 499, the protein expression was decreased in the L.T. gel. With respect to spot No. 531, the protein was only present in the control gel, however, with spots No. 701, 965, and 1292, the protein was only present in the L.T. gel.

## 4. Discussion

To date, despite increasing interest in, and the significance of exosomes, there are no standard procedures for their isolation, detection, and characterization. The reason for this is that exosomes are nanometers in size and are barely detected by conventional methods. Therefore, the goal of the present study was to effectively isolate U-87MG exosomes from cell culture medium and characterize them by protein analysis.

As shown in the TEM images, the pelleted exosomes were isolated successfully by filtration, concentration of the cell culture medium, and consecutive centrifugation, which confirmed the existence of exosomes. The most common protein present in exosomes, Hsp90, was detected by Western blotting. Hsp90 was increased in exosomes exposed to a low temperature compared with exosomes incubated under normal conditions. According to 2-DE images and MALDI-TOF-MS, different proteins were detected between the control gel and the L.T. gel. In the L.T. gel, certain proteins showed an increase in expression, whereas others showed a decrease. Moreover, there were spots that were only present on one of the gels. On the L.T. gel, there was increased expression of calcium-dependent secretion activator 2 isoform b, hCG1817425, armadillo repeat-containing protein 4, and immunoglobulin heavy variable 5-a, partial. The proteins that were reduced on the L.T. gel were collagen alpha-1(VI), DNA topoisomerase I, titin, mitochondrial isoform 2, RNA-binding protein 15B, phosphoserine aminotransferase isoform 2, and Chain A, Substrate Induced Remodeling Of The Active Site Regulates HTRA1 Activity. The protein spots that were present only on the control gel were coatomer protein complex, subunit beta 2 (isoform CRA_b), and hypothetical protein FLJ40243 (isoform CRA_a). Whereas the proteins spots that were present only in the L.T. gel were immunoglobulin gamma heavy chain variable region, nuclear receptor ROR-alpha isoform b, myosin-heavy chain 1, skeletal muscle, keratin-type I cytoskeletal 9, and Chain A, The Effect Of Metal Binding On The Structure Of Annexin V And Implications For Membrane Binding.

This study provides the first proteome analysis of U-87MG exosome and its changes in response to low temperature. Malign tumor marker protein such as human chorionic gonadotropin (hCG) was detected in U-87MG exosome and increased in response to cold temperature stimulus [37]. Among the proteins that were detected in U-87MG exosome, coatomer protein complex subunit, collagen type VI, myosin heavy chain 1, keratin, annexin A5 type I collagen, type VI alpha 1 are constituent structural proteins with omnipresent occurrence in cells and extracellular matrix. Coatomer is a soluble seven-protein complex which is a precursor of coat protein I (COP I; retrograde transport from trans-Golgi apparatus to cis-Golgi and endoplasmic reticulum) [38]. COP I plays a key role in Golgi apparatus vesicle formation, Golgi budding, and vesicular trafficking [38]. Collagen VI is a distinctive constituent within the superfamily of collagenous proteins and forms a main class of tissue microfibrils [39]. Annexin A5 which is a calcium- and phospholipid-binding protein interacts with a metastasis-related protein, polycystin-1 both in vitro and in a cell culture model and induces suppression of tumor metastasis [40]. It was also shown that polycystin-1 co-localizes with E-cadherin at the cell-cell contacts, accelerating the recruitment of intracellular E-cadherin to reforming junctions [40]. These exosome proteins could be proposed as targets for the detection of a cancer biomarker [41,42,43].

## 5. Conclusions

Altogether, the investigation of exosome proteome derived from the U-87MG glioblastoma cells, and the changes in metastasis marker proteins such as hCG and annexin A5 under temperature stress, suggest a possibility that these proteins may be used as diagnostic markers.

## Figures and Tables

**Table 1:** Exosomal proteins identified by MALDI-TOF-MS for each protein described in Figure 3.

(**A**) The proteins whose expression was increased in the L.T. gel.								
**Spot ID**	**Protein Description**	**Accession No.**	**Score**	**MW (Da)**	**pI**	**Coverage (%)**	**Matched Peptides No.**	**L.T./Ctr Ratio (% vol.)**
262	calcium-dependent secretion activator 2 isoform b	gi|148839284	54	144,652	5.80	13	14	2.0
444	hCG1817425	gi|119590387	68	15,559	8.88	63	7	3.2
859	armadillo repeat-containing protein 4	gi|31657114	53	117,146	7.98	17	13	2.6
1098	immunoglobulin heavy variable 5	gi|371571045	54	10,996	9.10	62	6	2.3
(**B**) The proteins whose expression was decreased in the L.T. gel.								
**Spot ID**	**Protein Description**	**Accession No.**	**Score**	**MW (Da)**	**pI**	**Coverage (%)**	**Matched Peptides No.**	**Ctr/L.T. Ratio (% vol.)**
91	collagen, type VI, alpha 1, isoform CRA_b	gi|119629727	76	109,602	5.26	24	19	3.6
282	DNA topoisomerase I, mitochondrial isoform 2	gi|386642871	56	59,178	9.18	26	10	3.4
293	titin	gi|407139	62	524,823	8.06	8	28	3.5
322	putative RNA-binding protein 15B	gi|54607124	66	97,432	9.86	21	13	2.2
496	phosphoserine aminotransferase isoform 2	gi|10863955	59	35,508	6.23	35	8	2.0
499	Chain A, Substrate Induced Remodeling Of The Active Site Regulates HTRA1 Activity	gi|323714490	101	36,607	6.91	44	12	2.1
(**C**) The protein spots that were only present in the control gel.								
**Spot ID**	**Protein Description**	**Accession No.**	**Score**	**MW (Da)**	**pI**	**Coverage (%)**	**Matched Peptides No.**	
531	coatomer protein complex, subunit beta 2 (beta prime), isoform CRA_b	gi|119599446	67	99,839	5.04	22	14	
536	hypothetical protein FLJ40243, isoform CRA_a	gi|119576413	51	183,085	6.14	12	17	
(**D**) The protein spots that were only identified in the L.T. gel.								
**Spot ID**	**Protein Description**	**Accession No.**	**Score**	**MW (Da)**	**pI**	**Coverage (%)**	**Matched Peptides No.**	
371	immunoglobulin gamma heavy chain variable region	gi|5051322	63	13,695	7.94	52	6	
372	nuclear receptor ROR-alpha isoform b	gi|19743901	61	64,306	5.97	15	10	
701	myosin, heavy chain 1, skeletal	gi|109731497	127	223,975	5.62	17	28	
701	muscle, adult	gi|109731497	127	223,975	5.62	17	28	
965	keratin, type I cytoskeletal 9	gi|55956899	89	62,255	5.14	29	12	
1292	Chain A, The Effect Of Metal Binding On The Structure Of Annexin V And Implications For Membrane Binding	gi|809185	112	35,840	4.94	38	14	
